# The prognostic significance of early blood neurofilament light chain concentration and magnetic resonance imaging variables in relapse‐onset multiple sclerosis

**DOI:** 10.1002/brb3.2700

**Published:** 2022-08-04

**Authors:** Thomas Williams, Amanda Heslegrave, Henrik Zetterberg, Katherine A Miszkiel, Frederik Barkhof, Olga Ciccarelli, Wallace J Brownlee, Jeremy Chataway

**Affiliations:** ^1^ Queen Square Multiple Sclerosis Centre, Department of Neuroinflammation, UCL Queen Square Institute of Neurology, Faculty of Brain Sciences University College London London UK; ^2^ National Hospital for Neurology and Neurosurgery Queen Square London UK; ^3^ Department of Neurodegenerative Disease UCL Institute of Neurology London UK; ^4^ UK Dementia Research Institute at UCL University College London London UK; ^5^ Department of Psychiatry and Neurochemistry Institute of Neuroscience and Physiology, the Sahlgrenska Academy at the University of Gothenburg Mölndal Sweden; ^6^ Clinical Neurochemistry Laboratory Sahlgrenska University Hospital Mölndal Sweden; ^7^ Centre for Medical Image Computing (CMIC), Department of Computer Science, Faculty of Engineering Sciences University College London London UK; ^8^ Radiology & Nuclear Medicine VU University Medical Centre Amsterdam The Netherlands; ^9^ Department of Brain Repair and Rehabilitation, UCL Queen Square Institute of Neurology, Faculty of Brain Sciences University College London London UK; ^10^ National Institute for Health Research University College London Hospitals Biomedical Research Centre London UK

**Keywords:** magnetic resonance imaging, multiple sclerosis, neurofilament light chain, prognosis

## Abstract

**Background:**

Improved prognostication remains vital in multiple sclerosis to inform personalized treatment approaches. Blood neurofilament light (bNfL) is a promising prognostic biomarker, but to what extent it provides additional information, independent of established MRI metrics, is yet to be established.

**Methods:**

We obtained all available bNfL data for 133 patients from a longitudinal observational cohort study. Patients were dichotomized into good or poor outcome groups based upon clinical and cognitive assessments performed 15 years after a clinically isolated syndrome. We performed longitudinal modeling of early NfL and MRI variables to examine differences between outcome groups.

**Results:**

The bNfL dataset was incomplete, with one to three (mean 1.5) samples available per participant. Within 3 months of onset, bNfL was similar between groups. The bNfL concentration subsequently decreased in those with a good outcome, and remained persistently elevated in those with a poor outcome. By year 5, NfL in the poor outcome group was approximately double that of those with a good outcome (14.58 [10.40–18.77] vs. 7.71 [6.39–9.04] pg/ml, respectively). Differences were reduced after adjustment for longitudinal changes in T2LV, but trends persisted for a greater rate of increase in NfL in those with a poor outcome, independent of T2LV.

**Conclusions:**

This analysis requires replication in cohorts with more complete bNfL datasets, but suggests that persistently elevated blood NfL may be more common in patients with a poor long‐term outcome. Persistent elevation of blood NfL may provide additional prognostic information not wholly accounted for by standard monitoring techniques.

## Graphical abstract

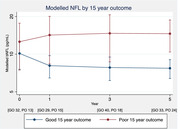

## 1 INTRODUCTION

The prognosis of multiple sclerosis (MS) is highly variable, with important implications for the management of patient expectations and clinical decision making around disease‐modifying therapies (DMTs). While randomized controlled trial data on whether all patients with relapsing remitting MS (RRMS) should be offered high‐efficacy DMT first line is awaited, most clinicians use demographic, clinical, and MRI variables to personalize treatment plans (Ontaneda et al., 2019). Data from historic, largely untreated cohort studies remain vital to this process (Brownlee et al., 2019, Tintore et al., 2015).

Early studies into patients with relapse‐onset MS identified clinical features associated with poor long‐term prognosis (Scalfari et al., 2014, Eriksson & Andersen, 2003, Confavreux et al., 2003). The inclusion of longitudinal MRI variables subsequently expanded our ability to predict disability outcomes, with evidence of ongoing lesion accumulation, particularly when located in clinically eloquent sites, being of central importance (Brownlee et al., 2019, Tintore et al., 2015, O'Riordan et al., 1998, Brex et al., 2002, Tintoré et al., 2006, Fisniku et al., 2008, Swanton et al., 2009, Di Filippo et al., 2010, Tintore & Castillo, 2010). The rate of change in T2 lesion volume (T2LV) from baseline to 5 years is associated with 20 year Expanded Disability Status Scale (EDSS) outcomes (*r* ^2 ^= 0.61 [0.43–0.74]), and the development of new spinal cord or infratentorial lesions from baseline to 3 years significantly increases the odds of developing secondary progressive MS (SPMS) at 15 years (spinal cord lesions: OR 38.68 [4.67–320.53]; infratentorial lesions: OR 3.28 [0.87–12.31]). (Brownlee et al., 2019, Fisniku et al., 2008)

Until recently, fluid biomarkers of prognosis were limited to cerebrospinal fluid (CSF) oligoclonal bands (Tintore et al., 2015, Dobson et al., 2013). Our ability to quantify neurofilament light chain (NfL), as a fluid biomarker of neuroaxonal injury in either CSF or blood, has led to numerous studies assessing the prognostic significance of CSF NfL (cNfL) or blood NfL (bNfL).

Higher baseline cNfL appears to be associated with current active or chronic neuroinflammation, future inflammatory disease activity, and worse long‐term disability outcomes (Ferreira‐Atuesta et al., 2021, Maggi et al., 2021, Bhan et al., 2018, Modvig et al., 2014, Salzer et al., 2010, Kuhle et al., 2020, Ferraro et al., 2016, Kuhle et al., 2017). In one mixed cohort of patients with relapsing remitting (RR) or progressive MS (PMS), a 1000 pg/ml increase in baseline cNfL was associated with a subsequent EDSS increase of 0.47 [0.25–0.69] points over the next 5 years (Bhan et al., 2018). This has been replicated in some cohorts for bNfL, though the comparative ease with which bNfL can be repeatedly sampled has facilitated demonstrations that persistently elevated bNfL may have more prognostic significance than baseline measures alone (Kuhle et al., 2018, Kuhle et al., 2018, Kuhle et al., 2019, Kuhle et al., 2017, Calabresi et al., 2018, Cantó et al., 2019, Friedova et al., 2020). In one large mixed cohort, patients with subsequent EDSS worsening over 10 years were more likely to experience increases in bNfL compared with those with a stable EDSS (worsening EDSS: 1.017 pg/ml/year increase; stable EDSS: 1.002 pg/ml/year increase, *p* < .001), while baseline bNfL levels were similar (21.8 vs. 21.3 pg/ml, *p* = .69) (Cantó et al., 2019).

A weakness of the existing literature is that when assessing the prognostic significance of bNfL, few studies take into account established MRI prognostic variables already available in clinical practice, such as T2 lesion load, location and activity. Those that do include MRI covariates often only include baseline variables, when longitudinal changes are known to be more informative (Plavina et al., 2020, Barro et al., 2018). It is yet to be established whether longitudinal measures of bNfL add independently significant prognostic information to patients following their first demyelinating event, or whether it is merely reinforcing what is established with MRI data. As bNfL approaches use in clinical practice, this will soon become an important question for those making treatment decisions in early RRMS (Leppert & Kuhle, 2019).

Here, we obtained all available bNfL data from an existing prospective, longitudinal, observational cohort of patients with relapse‐onset MS and 15 years of follow‐up. We modeled bNfL together with lesional and volumetric MRI variables over the first 5 years from clinical onset, based upon a long‐term clinical outcome assessed 15 years after disease onset, to investigate the relationship of bNfL and MRI with disease course in the longer term.

## 2 METHODS

### 2.1 Participants

Participants were prospectively recruited as previously reported (Brownlee et al., 2019). Briefly, between 1995 and 2004, patients with a clinically isolated syndrome (CIS) suggested of MS were enrolled in a longitudinal clinical and MRI study. Baseline clinical and MRI assessments were completed within 3 months of onset, and repeated at 1, 3, 5, and 15 years. At the final follow‐up timepoint, multiple aspects of MS‐related disability were assessed including EDSS, timed 25‐foot walk (25FW), 9‐hole peg test (9HPT), and cognitive assessments including paced auditory serial addition test (3 s; PASAT3) and the symbol digit modality test (SDMT).

### 2.2 MRI acquisition and analysis

From baseline to 5 years, all participants underwent the same MRI protocol on the same 1.5T Signa scanner, as previously described (Brownlee et al., 2019). Briefly, axial proton‐density (PD)/T2‐weighted and post‐contrast T1‐weighted fast‐spin echo scans of the brain were acquired. Spinal cord MRI included sagittal T2‐weighted and post‐contrast T1‐weighted scans of the whole spine and a volume acquired inversion prepared fast spoiled gradient echo scan of the cervical cord. All scans were reviewed by an experienced neuroradiologist who performed counts of PD/T2 and post‐contrast T1 lesions. T2 lesion volume (T2LV) and T1 lesion volume (T1LV) was calculated from lesion masks obtained using a semi‐automated edge‐finding tool (JIM6.0; Xinapse Systems, Aldwincle, UK) with manual correction. The 2D T1‐weighted fast‐spin echo scans were used for the volumetric brain measures. Using the baseline MRI scan, the normalized brain volume was calculated using SIENAX and follow‐up scans were registered with the baseline MRI scan to calculate the percentage brain volume change (PBVC) over time using SIENA (Smith et al., 2002). Upper cervical cord area was calculated as previously described using a active surface model and percentage change with time determined by reference to the baseline scan (Brownlee et al., 2019, Brownlee et al., 2017).

### 2.3 NfL data

Blood samples were not systematically collected as part of the original study protocol, but 133 of the 166 participants with longitudinal follow‐up had at least one plasma or serum samples available for analysis. A mean of 1.5 samples (range 1–3) were available for each participant in the study, with sample availability well balanced between those defined as having a good or poor outcome (Table 1). Most samples analyzed were plasma. As serum and plasma NfL are strongly correlated in RRMS (*r* = 0.89–0.96), and the proportion of plasma and serum samples were well balanced between the two outcome groups (Table 1), we included all plasma and serum samples in the analysis without adjustment (Hendricks et al., 2019, Sejbaek et al., 2019).

**TABLE 1:** Descriptive statistics for the patients included in this analysis

Variable	Frequency in analyzed cohort
*N*	133
Female	64%
Age at onset (mean)	32.8 (SD 7.6, range 16.6–50.9)
Follow‐up duration (mean)	14.3 (SD 3.3)
Syndrome at onset	114 ON, 4 SC, 14 BS, 1 HS
Baseline EDSS (median)	1.0, IQR 1 to 2, range 0–4
Baseline T2LV (median)	0.41 mL, IQR 0.05 to 1.46
15‐year EDSS (median)	1.5, IQR 1 to 3, range 0‐10
15‐year T2LV (median)	3.0 mL, IQR 0.65–9.60
CDMS	59%
McDonald 2017 MS	73%
DMT during follow‐up (any time)	29 (21.8%)
DMT prior to first NFL sample	6 (4.5%)
Total bNfL samples analyzed Baseline Year 1 Year 3 Year 5	204 45 (good outcome 32; poor outcome 13) 44 (good outcome 29; poor outcome 15) 58 (good outcome 40; poor outcome 18) 57 (good outcome 33; poor outcome 24)
bNfL samples by outcome group	Good outcome: 1.58 samples per patient, 83.6% plasma Poor outcome: 1.46 samples per patient, 90.0% plasma
Defined as poor outcome	48 (36%)
Contributions to poor outcome definition:	
SPMS	18 (14%)
EDSS >= 3.0	33 (25%)
SDMT *Z*‐score < −1.5	19 (14%)
PASAT3 *Z*‐score < −1.5	17 (13%)
25FW *Z*‐score < −1.5	9 (7%)
9HPT *Z*‐score < −1.5	10 (8%)

ON, optic neuritis; SC, spinal cord; BS, brainstem; HS, hemispheric; EDSS, Expanded Disability Status Score; T2LV, T2 lesion volume; CDMS, clinically definite multiple sclerosis; DMT, disease‐modifying therapy; bNfL, blood neurofilament light; SPMS, secondary progressive multiple sclerosis; SDMT, symbol digit modality test; PASAT3, paced auditory serial addition test; 25FW, timed 25‐foot walk test; 9HPT, 9‐hole peg test.

Samples were collected at the time of clinical assessments and stored at −80°C until the time of analysis. All samples were analyzed in duplicate using the Quanterix Simoa NF‐light advantage assay on a HD‐1 Analyser (Quanterix, Billerica, MA, USA). Recent reports have highlighted the benefits of analyzing NfL as age‐ and body mass index (BMI)‐adjusted *Z*‐scores (Benkert et al., 2022). Since access to the required control and BMI data were not available for this historical cohort, we analyzed NfL without adjustment, but included age as a covariate in all multivariable analyses.

### 2.4 Statistical analysis

#### 2.4.1 Categorical definition of long‐term clinical outcome

To more fully capture the multifaceted aspects of long‐term disability prioritized by patients with MS, we defined patients as having a poor long‐term outcome if at their final assessment, any one or more of the following features were present: diagnosis of SPMS; EDSS > = 3.0; cognitive impairment defined by PASAT3 or SDMT *Z*‐score < ‐1.5; motor impairment defined by 25FW or 9HPT *Z*‐score < ‐1.5. *Z*‐scores were calculated based upon age‐matched normative data.

#### 2.4.2 Longitudinal modeling of early NfL or imaging predictors between outcome groups

A statistical analysis plan was formulated prior to undertaking longitudinal modeling. Linear mixed models were used in order to include all patients with at least one bNfL sample in the analysis, retaining the timepoint since clinical onset at which the bNfL sample was taken. For the prespecified primary analysis, the dependent variable was bNfL from baseline to 5 years from clinical onset. The independent fixed effect variable was the categorical definition of good or poor long‐term outcome. An interaction between outcome and time (categorically defined) was included. A random effect was included at the level of the participant. Marginal means and their 95% confidence interval were then calculated from this model to produce estimates for the bNfL concentration at each timepoint in the good and poor outcome groups from a single model. A separate model was also constructed with time as a continuous variable, to produce estimates of the difference in the overall rate of change in bNfL from baseline across all timepoints between the two outcome groups. Age at clinical onset, and its interaction with time, was additionally included as a fixed effect covariate in all analyses due to its established impact upon bNfL (Benkert et al., 2022).

The prespecified secondary analysis was to repeat the above modeling, but with the inclusion of longitudinal MRI variables currently used in clinical practice (such as T2 lesion load), and their interaction with time, as covariates to assess the extent by which bNfL differs between the two outcome groups after adjusting for longitudinal changes in MRI variables. Exploratory analyses included repeating the primary analysis, but with longitudinal MRI variables, such as T2LV, T1LV, spinal cord lesions, T1‐GAD+ lesions, and the rate of whole brain or cervical cord atrophy as the dependent variable.

For all models, estimates were generated through nonparametric, bias‐corrected and accelerated bootstrap with 10,000 replications due to violations of parametric model assumptions. *p* Values are therefore not calculated but may be inferred from the confidence intervals of the estimates. Additionally, an unstructured residual covariance matrix was included, allowing a different variance at each visit and different covariances between each pair of measurements on the same participant, where repeated measures are available for the prognostic variables.

#### 2.4.3 Ethical approval and consent

The original prospective cohort study was approved by the institutional ethics committees at the National Hospital for Neurology and Neurosurgery and Moorfields Eye Hospital. The 15‐year follow‐up study was approved by the National Research Ethics Service (NRES) Committee London (City Rd and Hampstead). Written informed consent was obtained from study participants at the time of study entry and at each subsequent follow‐up visit. Approval for reanalysis of stored patient blood samples for NfL was obtained through the NRES (London – Queen Square Research Ethics Committee). All research was conducted in accordance to the declaration of Helsinki. (World Medical Association)

#### 2.4.4 Data availability

The data that support the findings of this study are available from the corresponding author upon reasonable request.

## 3 RESULTS

Descriptive statistics for the 133 participants with available bNfL data are shown in Table 1. Six patients (4.5%) received DMT prior to the first bNfL sampling (beta interferon or glatiramer acetate in all cases). For the 29 patients (21.8%) receiving DMT at any point during follow‐up, the majority received beta interferon or glatiramer acetate, with nine patients (6.8%) escalating to second line therapies. 36% were defined as having a poor long‐term outcome. Of the nine patients in the poor outcome group with EDSS >= 3 at baseline, all either continued to experience EDSS worsening, or improved on EDSS but had impaired cognition at 15 years.

### 3.1 Cross‐sectional comparisons between bNfL, MRI, and clinical variables

**FIGURE 1:**
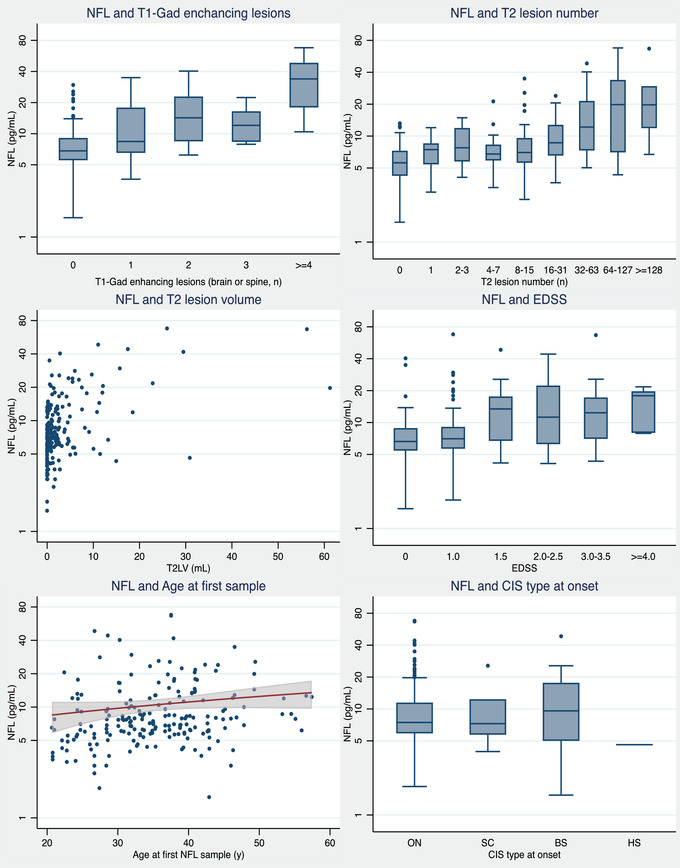
Cross‐sectional comparisons between NfL and clinical and MRI variables at all timepoints. bNfL, blood neurofilament light; EDSS, Expanded Disability Status Scale; CIS, clinically isolated syndrome; ON, optic neuritis; SC, spinal cord; BS, brainstem; HS, hemispheric

As shown in Figure 1, the expected cross‐sectional relationships were apparent between bNfL and MRI/clinical variables when viewed across all timepoints.

### 3.2 Longitudinal changes in bNfL between good and poor outcome groups

**FIGURE 2:**
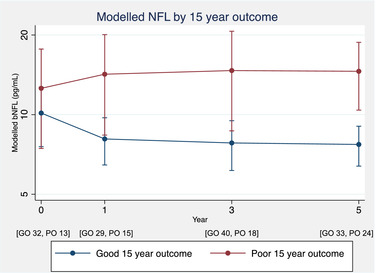
Early longitudinal NfL modeling by 15‐year outcome groups. Modeled longitudinal bNfL data comparing the marginal means and their 95% confidence intervals between the good and poor outcome groups, estimated from a single model based upon distributions generated from 10,000 bootstrap replications. Age at clinical onset, and its interactions with time, is included as a covariate. The number of available bNfL samples in each outcome group at each timepoint is included below the x‐axis. bNfL, blood neurofilament light; GO, good outcome; PO, poor outcome

Modeling of bNfL between outcome groups is summarized in Table 2 and Figure 2. At baseline (within 3 months of clinical onset), the differences in bNfL between outcome groups were small and not statistically significant. Patients with a good clinical outcome, however, tended to demonstrate a reduction in bNfL after the first demyelinating event, which remained persistently low throughout the next 5 years. In contrast, there was a trend for those in the poor outcome group to experience a greater rate of increase in bNfL with time (0.72 [−0.58 to 3.29] pg/ml/year greater, compared with the good outcome group), such that by 5 years after clinical onset, patients with a poor outcome had a significantly higher bNfL, approaching double that seen in those with a good clinical outcome (good outcome: 7.71 [6.39–9.04] pg/ml; poor outcome: 14.58 [10.40–18.77] pg/ml).

**TABLE 2:** Marginal means and 95% confidence intervals of bNfL and MRI variables between 15‐year good and poor outcome groups

Dependent variable	15‐year outcome group	Baseline(pg/ml)	1 year(pg/ml)	3 years(pg/ml)	5 years(pg/ml)	Difference in rate of change between outcome groups (unit/year)
bNfL (pg/ml) [95% CI]	Good outcome	10.14 [7.57–12.71]	8.09 [6.45–9.73]	7.82 [6.15–9.48]	7.71 [6.39–9.04]	0.72 [−0.58 to 3.29]
	Poor outcome	12.57 [7.45–17.70]	14.22 [8.37–20.07]	14.68 [8.68–20.67]	14.58 [10.40–18.77]	
T2LV (ml) [95% CI]	Good outcome	0.87 [0.48–1.27]	1.25 [0.65–1.84]	1.61 [0.90–2.32]	1.91 [1.17–2.65]	1.23 [0.67–2.12]
	Poor outcome	2.85 [1.48–4.21]	5.11 [3.03–7.20]	5.95 [3.75–8.15]	10.68 [7.04–14.33]	
T1LV (ml) [95% CI]	Good outcome	0.10 [0.02–0.17]	0.15 [0.07–0.24]	0.20 [0.09–0.31]	0.27 [0.11–0.43]	0.38 [0.15–1.09]
	Poor outcome	0.27 [−0.09–0.63]	0.65 [0.23–1.08]	0.96 [0.45–1.48]	2.51 [0.58–4.45]	
Cord lesions (*n*) [95% CI]	Good outcome	0.37 [0.19–0.55]	0.55 [0.34–0.76]	0.77 [0.47–1.07]	0.95 [0.61–1.29]	0.41 [0.22–0.65]
	Poor outcome	1.31 [0.82–1.79]	1.40 [0.89–1.91]	2.54 [1.88–3.20]	3.85 [2.82–4.88]	
T1‐GAD+ (*n*) [95% CI]	Good outcome	0.43 [0.13–0.72]	0.35 [0.13–0.57]	0.32 [0.13–0.51]	0.13 [−0.07 to 0.34]	−0.25 [−0.68 to 0.02]
	Poor outcome	2.37 [1.27–3.47]	1.98 [0.40–3.56]	1.55 [0.09–3.01]	0.78 [−0.16 to 1.71]	
PBVC (%) [95% CI]	Good outcome	NA	−0.23 [−0.33 to −0.13]	−0.80 [−0.97 to −0.64]	−1.84 [−2.29 to −1.39]	−0.28 [−0.48 to −0.12]
	Poor outcome	NA	−0.45 [−0.64 to −0.26]	−1.46 [−1.90 to −1.02]	−3.23 [−3.96 to −2.50]	
UCCA‐PC (%) [95% CI]	Good outcome	NA	−0.21 [−0.54 to 0.12]	−0.60 [−0.93 to −0.27]	−1.55 [−2.10 to −0.10]	−0.50 [−0.89 to −0.21]
	Poor outcome	NA	−0.90 [−1.59 to −0.22]	−2.05 [−2.85 to −1.24]	−4.15 [−5.76 to −2.54]	
bNfL (pg/ml) adjusted for T2LV [95% CI]	Good outcome	14.26 [4.68–23.83]	11.94 [8.95–14.92]	10.30 [6.90–13.70]	8.01 [6.39–9.63]	0.63 [−0.30 to 3.39]
	Poor outcome	14.22 [7.12–21.31]	11.75 [6.38–17.12]	11.58 [7.18–15.97]	10.86 [6.82–14.91]	

Marginal means and 95% confidence intervals for bNfL and MRI variables at each timepoint in the good and poor outcome groups, estimated from a single model based upon distributions generated from 10,000 bootstrap replications. The overall difference in the rate of change of each dependent variable from baseline to 5 years (plus bias‐corrected and accelerated 95% confidence intervals) from a separate model with time as a continuous variable is also reported in the final column. For PBVC and UCCA‐PC, as these measures are reported as the % change from baseline, no data is reported for the baseline timepoint. bNfL, blood Neurofilament light; NA, Not Applicable; T2LV, T2 lesion volume; T1LV, T1 lesion volume; GAD, Gadolinium; PBVC, percentage whole brain volume change; UCCA‐PC, Upper cervical cord area percentage change.

The analysis was repeated for the subgroup of patients who were diagnosed with MS during follow‐up (97 patients; 47% having a poor outcome). The results were similar to those from the whole cohort (Table S1 and Figure S1). At baseline, NfL was similar between the good and poor outcome groups (11.49 [7.43–15.56] pg/ml vs. 12.88 [7.53–18.22] pg/ml, respectively), but the trend for a greater rate of increase in NfL from baseline to 5 years (0.86 [−0.62 to 3.56] pg/ml/year greater in the poor outcome group), resulted in a significantly higher NfL at 5 years in the poor outcome group (good outcome: 8.46 [6.45–10.47] pg/ml vs. poor outcome: 15.63 [11.01–20.26] pg/ml). The results were also similar when including adjustment for bNfL sample type (plasma or serum), and when analyses were run only including the 175 plasma NfL samples (Table S2).

### 3.3 Longitudinal changes in MRI variables between good and poor outcome groups

**FIGURE 3:**
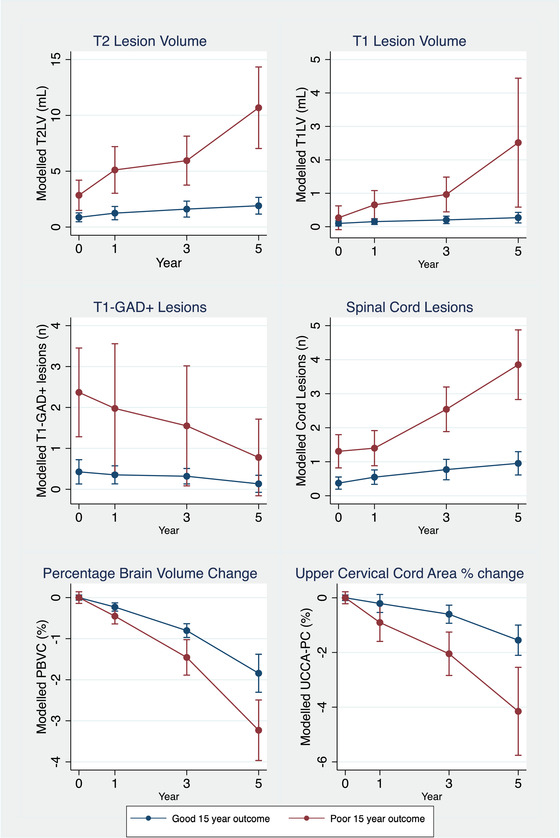
Early longitudinal MRI modeling by 15‐year outcome groups. Modeled longitudinal lesional and volumetric MRI data comparing the marginal means and their 95% confidence intervals between the good and poor outcome groups, estimated from a single model based upon distributions generated from 10,000 bootstrap replications. For both PBVC and percentage upper cervical cord area change, the baseline timepoint acts as the reference, and is set to zero. Age at clinical onset, and its interactions with time, is included as a covariate. T1‐GAD+, T1 post‐contrast enhancing lesions; PBVC, percentage whole brain volume change; UCCA‐PVC, upper cervical cord area percentage change. T2LV, T2 lesion volume; T1LV, T1 lesion volume

Modeling of MRI variables between outcome groups is summarized in Table 2 and Figure 3. While T2LV was modestly higher in the poor outcome group at baseline, the greater rate of increase in T2LV (1.23 [0.67–2.12] ml/year higher) resulted in greater T2LVs accumulating with time, such that by 5 years after CIS, the poor outcome group had a mean T2LV greater than five times the good outcome group (good outcome: 1.91 [1.17–2.65] ml; poor outcome: 10.68 [7.04–14.33] ml). Similar results were seen for the modeling of spinal cord lesions and T1LV. The volumetric measures of whole brain atrophy and upper cervical cord atrophy (PBVC and UCCA‐PC) both demonstrated significantly greater rates of volume loss in those with a poor long‐term outcome.

### 3.4 Longitudinal changes in bNfL while adjusting for T2 lesion volume

**FIGURE 4:**
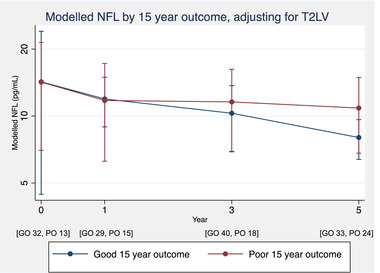
Longitudinal modeling of bNfL while adjusting for T2 lesion volume, between good and poor outcome groups. Modeled longitudinal bNfL data comparing the marginal means and their 95% confidence intervals between the good and poor outcome groups, estimated from a single model based upon distributions generated from 10,000 bootstrap replications. Both intracranial T2 lesion volume and age at clinical onset, and their interactions with time, are included as covariates. The number of available bNfL samples in each outcome group at each timepoint is included below the x‐axis. bNfL, blood neurofilament light; GO, good outcome; PO, poor outcome

Current guidance recommends the use of brain MRI only in the radiological monitoring of patients with MS (Wattjes et al., 2015). We therefore focused on longitudinal changes in bNfL while adjusting for brain T2LV, as summarized in Table 2 and Figure 4. After adjusting for longitudinal changes in T2LV, the previously described differences in NfL between the good and poor outcome groups were attenuated. In particular, the marginal means and 95% confidence intervals at baseline and 1 year became very similar between the two groups, suggesting the variability in bNfL between the two outcome groups may largely be accounted for by changes in T2LV. At 3‐ and 5‐year timepoints, the confidence intervals were wide and overlapping between the two outcome groups. The marginal means, however, particularly at 5 years, were higher in the poor outcome group, and the overall rate of change in bNfL between groups, across all timepoints, again suggested a trend to a greater rate of increase in the poor outcome group (0.63 [−0.30 to 3.39] pg/ml/year greater increase in the poor outcome group). Adjusting for additional MRI covariates did not substantially alter these results.

## 4 DISCUSSION

Our results suggest that bNfL concentrations, within 3 months of disease onset, are similar between those with a good and poor long‐term outcome. Those with a good long‐term outcome, however, tend to subsequently experience a gradual and persistent reduction in bNfL, while bNfL tends to increase and remain persistently elevated in those with a poor outcome. At 5 years from disease onset, those with a poor outcome have a significantly higher bNfL, estimated to be almost double that of those with a good outcome.

When adjusting for change in T2LV, the differences in bNfL between prognostic groups were reduced. At baseline and 1 year after clinical onset, adjusted bNfL is similar in those with good and poor long‐term outcomes. The trend toward a greater rate of increase in bNfL in the poor outcome group, however, persists despite adjustment for T2LV, suggesting that independent of changes in T2LV, a persistently elevated bNfL may be of prognostic importance.

The key limitation of this work is the incomplete availability of bNfL samples. This introduces the potential for bias and reduces our power to detect differences in bNfL between the outcome groups, although importantly, the number of NfL samples was well balance between outcome groups. Our analyses should therefore be viewed as preliminary, should only be interpreted at the group level, and require confirmation in similar prospective cohorts with standardized collection bNfL, as well as clinical and MRI data. While we therefore conclude that from our current data, there is insufficient evidence to support a difference in bNfL between the outcome groups beyond that accounted for by variability in T2LV, we may be underpowered to detect small differences. Certainly, the trend toward a greater rate of increase in bNfL in those with a poor long‐term outcome, despite adjusting for changes in T2LV, suggests that further studies are warranted to confirm if persistently elevated bNfL does contribute additional prognostic information beyond that obtained through our current clinical practice of routine monitoring of intracranial T2 lesion burden. Additionally, we cannot exclude the possibility of significant differences being found in bNfL between prognostic groups, independent of T2LV, at earlier timepoints if more bNfL data were available.

Our results are largely in keeping with those of Cantó et al., who found that in a mixed cohort of pwMS, patients experiencing EDSS progression were more likely to demonstrate increases in sNfL with time compared with those that remained stable, while baseline sNfL was similar between groups (Cantó et al., 2019). In other CIS cohorts, however, early sNfL was prognostic for subsequent MS diagnosis after a median of 8.3 years (Dalla Costa et al., 2019). While this analysis by Dalla Costa et al. differs from our own, we would also suggest that the timing of the first NfL sample may contribute to our somewhat divergent results. All our baseline samples were obtained within 3 months of onset, with a median duration of 39 days from symptoms onset to blood sampling. Most included in the Dalla Costa et al. cohort were obtained >2 months from symptom onset (Dalla Costa et al., 2019). Their results may therefore actually be consistent with our own, in that persistent elevations of NfL may be of more prognostic significance than very early NfL levels following CIS onset. Further analysis in CIS cohorts with more complete NfL datasets, however, are required to assess the prognostic significant of early baseline NfL compared to the subsequent rate of change in sNfL.

Our finding of a trend toward a greater rate of increase in bNfL, independent of T2LV, in the poor prognostic group is supported by other recent studies. In a large mixed cohort of pwMS, and in a separate RRMS group, NfL appeared to provide additional prognostic information regarding future inflammatory disease activity, in addition to that determined through the monitoring of clinical and MRI variables (new T2 lesions or enhancing lesions) (Benkert et al., 2022, Uher et al., 2021). While one should be cautious about applying such group‐level data to individual patients, these results suggest that incorporating NfL monitoring into clinical practice may be useful in improving our ability to predict future disease activity.

The majority of patients included in this study never received DMT (78% untreated). While this was in accordance with UK practice at the time, most would now be offered treatment. The prognostic utility of longitudinal bNfL monitoring in clinical practice should also be examined in patients receiving immunomodulatory treatment in order to assess whether failure to normalize bNfL following initiation of treatment is associated with a poor long‐term prognosis. Early evidence from randomized controlled trial datasets suggests that persistently elevated NfL following initiation of treatment may indeed be associated with a worse outcome, although in patients with low levels of inflammatory activity on stable high‐efficacy DMT, progression appears to occur largely independent of baseline or longitudinal NfL concentrations (Kuhle et al., 2019, Bridel et al., 2021). Future studies should additionally assess whether persistently elevated NfL after treatment initiation should be included as a criteria for treatment escalation.

## 5 CONCLUSION

bNfL is similar between long‐term prognostic groups within 3 months of a first demyelinating event, with a subsequent persistent elevation of bNfL seen in those with a poor long‐term clinical outcome. Such differences are reduced after adjusting for changes in T2LV, but trends toward a greater rate of increase in bNfL persist, independent of T2LV, in those with a poor outcome. The incomplete bNfL dataset means this analysis is likely to be underpowered to detect smaller difference in bNfL between outcome groups, and the results should therefore be viewed as preliminary. Further studies with more complete datasets should look to confirm whether persistently elevated bNfL, the monitoring of which may soon be available in clinical practice, provides additional prognostic information beyond that obtained through the current clinical practice of routine monitoring of brain T2 lesion load.

## FUNDING

No specific funding was obtained for this research. T. W. in funded by the MS‐STAT2 clinical trial, with funding derived from NIHR Health Technology Assessment (HTA) Programme, UK Multiple Sclerosis Society, US National Multiple Sclerosis Society, and the Rosetrees Trust. The original prospective cohort study upon which this analysis is based was funded by the UK MS Society (grant number 995) and the Neurological Foundation of New Zealand (grant number 1207‐CF).

### PEER REVIEW

The peer review history for this article is available at https://publons.com/publon/10.1002/brb3.2700

## Supporting information

Figure S1: Early longitudinal NfL modelling by 15 year outcome groups in the subgroup of participants who developed multiple sclerosis

Table S1: Marginal means and 95% confidence intervals of bNfL between 15 year Good and Poor Outcome Groups in the subgroup of participants who developed multiple sclerosis

Table S2: Marginal means and 95% confidence intervals of bNfL between 15 year Good and Poor Outcome Groups whilst adjusting for sample type.
Table S3: Marginal means and 95% confidence intervals of plasma NfL between 15 year Good and Poor Outcome Groups.
