# Supplementary figures and images for: The prognostic significance of early blood neurofilament light chain concentration and magnetic resonance imaging variables in relapse‐onset multiple sclerosis
Source: Brain Behav. 2022 Aug 4;12(9):e2700. doi: 10.1002/brb3.2700 (PMC9480937; doi:10.1002/brb3.2700)

## Modelled NFL by 15 year outcome in patients diagnosed with MS

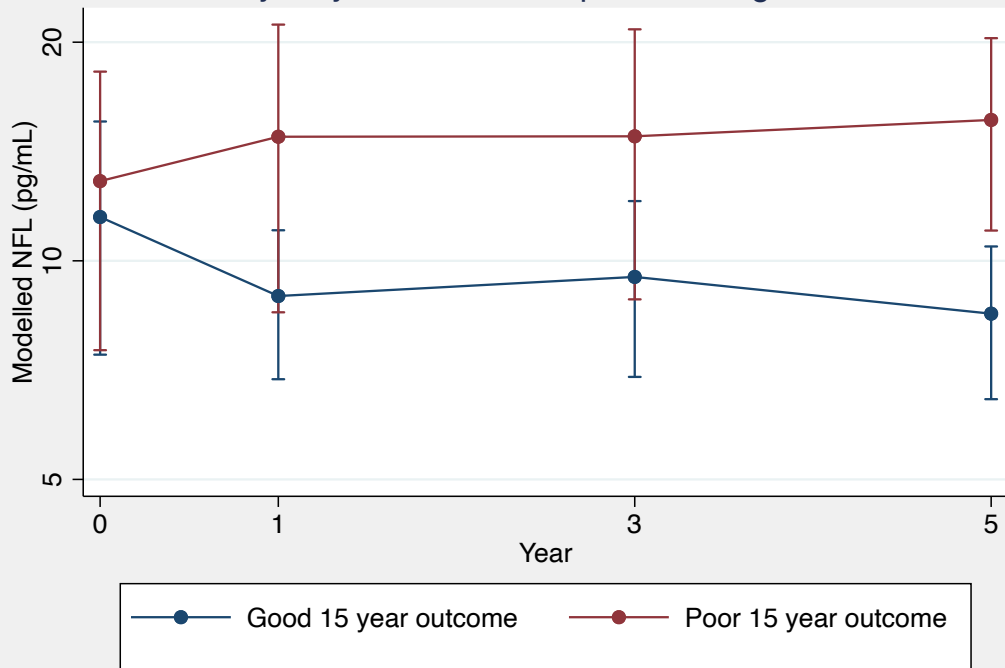

Supplement: Supplementary file 1 — Figure S1: Early longitudinal NfL modelling by 15 year outcome groups in the subgroup of participants who developed multiple sclerosis [file BRB3-12-e2700-s003.pdf]
