# Supplementary material for: The prognostic significance of early blood neurofilament light chain concentration and magnetic resonance imaging variables in relapse‐onset multiple sclerosis
Source: Brain Behav. 2022 Aug 4;12(9):e2700. doi: 10.1002/brb3.2700 (PMC9480937; doi:10.1002/brb3.2700)
Supplement: Supplementary file 2 — Table S1: Marginal means and 95% confidence intervals of bNfL between 15 year Good and Poor Outcome Groups in the subgroup of participants who developed multiple sclerosis [file BRB3-12-e2700-s002.docx]

| **Dependent variable** | **15 year outcome group** | **Baseline**  **(pg/mL)** | **1 year**  **(pg/mL)** | **3 years**  **(pg/mL)** | **5 years**  **(pg/mL)** | **Difference in rate of change between outcome groups (unit/year)** |
| --- | --- | --- | --- | --- | --- | --- |
| **bNFL (pg/mL)**  **[95% CI]** | Good Outcome | 11.49  [7.43 to 15.55] | 8.94  [6.87 to 11.02] | 9.50  [6.92 to 12.09] | 8.46  [6.45 to 10.47] | 0.855  [-0.62 to 3.60] |
|  | Poor Outcome | 12.88  [7.53 to 18.22] | 14.82  [8.49 to 21.15] | 14.84  [8.85 to 20.83] | 15.63  [11.01 to 20.26] |  |

Table S1: Marginal means and 95% confidence intervals of bNFL between 15 year Good and Poor Outcome Groups in the subgroup of participants who developed multiple sclerosis

Marginal means and 95% confidence intervals for bNFL at each timepoint for patients diagnosed with multiple sclerosis (McDonald 2017, n=97) during follow-up, in the good (n=51) and poor (n=46) outcome groups. In the good outcome group, 1.6 NfL samples were available per participant; in the poor outcome group, 1.5 NfL samples per participant. At each timepoint, the following number of bNFL samples were included: baseline (good outcome = 20, poor outcome = 13); 1 year (good outcome = 22, poor outcome = 14); 3 years (good outcome = 24, poor outcome = 18); 5 years (good outcome = 17, poor outcome = 22). Estimates from a single model based upon distributions generated from 10,000 bootstrap replications. The overall difference in the rate of change of each dependent variable from baseline to 5 years (plus bias-corrected and accelerated 95% confidence intervals) from a separate model with time as a continuous variable is also reported in the final column. bNFL, blood Neurofilament light; NA, Not Applicable; T2LV, T2 lesion volume; T1LV, T1 lesion volume; GAD, Gadolinium; PBVC, percentage whole brain volume change; UCCA-PC, Upper cervical cord area percentage change.
