# Supplementary material for: The prognostic significance of early blood neurofilament light chain concentration and magnetic resonance imaging variables in relapse‐onset multiple sclerosis
Source: Brain Behav. 2022 Aug 4;12(9):e2700. doi: 10.1002/brb3.2700 (PMC9480937; doi:10.1002/brb3.2700)
Supplement: Supplementary file 3 — Table S2: Marginal means and 95% confidence intervals of bNfL between 15 year Good and Poor Outcome Groups whilst adjusting for sample type. Table S3: Marginal means and 95% confidence intervals of plasma NfL between 15 year Good and Poor Outcome Groups. [file BRB3-12-e2700-s001.docx]

Table S2: Marginal means and 95% confidence intervals of bNFL between 15 year Good and Poor Outcome Groups whilst adjusting for sample type.

| Dependent variable | 15 year outcome group | Baseline  (pg/mL) | 1 year  (pg/mL) | 3 years  (pg/mL) | 5 years  (pg/mL) | Difference in rate of change between outcome groups (unit/year) |
| --- | --- | --- | --- | --- | --- | --- |
| bNFL (pg/mL)  [95% CI] | Good Outcome | 10.15  [7.55 to 12.76] | 8.01  [6.34 to 9.69] | 7.76  [6.10 to 9.41] | 7.83  [6.40 to 9.26] | 0.74  [-0.60 to 3.25] |
|  | Poor Outcome | 12.55  [7.62 to 17.49] | 14.16  [8.26 to 20.05] | 14.75  [8.69 to 20.82] | 14.72  [10.60 to 18.85] |  |
| bNFL (pg/mL) adjusted for T2LV  [95% CI] | Good Outcome | 14.36  [4.50 to 24.22] | 11.83  [8.75 to 14.92] | 10.26  [6.90 to 13.62] | 8.13  [6.37 to 9.88] | 0.64  [-0.29 to 3.00] |
|  | Poor Outcome | 14.22  [6.89 to 21.58] | 11.75  [6.39 to 17.08] | 11.58  [7.26 to 15.94] | 10.86  [7.06 to 14.92] |  |

Marginal means and 95% confidence intervals for bNFL at each timepoint in the good and poor outcome groups, whilst including a fixed-effect variable for sample type (plasma or serum). Estimates from a single model based upon distributions generated from 10,000 bootstrap replications. The overall difference in the rate of change of each dependent variable from baseline to 5 years (plus bias-corrected and accelerated 95% confidence intervals) from a separate model with time as a continuous variable is also reported in the final column. bNFL, blood Neurofilament light; T2LV, T2 lesion volume.

Table S3: Marginal means and 95% confidence intervals of the 175 plasma NFL between 15 year Good and Poor Outcome Groups.

| Dependent variable | 15 year outcome group | Baseline  (pg/mL) | 1 year  (pg/mL) | 3 years  (pg/mL) | 5 years  (pg/mL) | Difference in rate of change between outcome groups (unit/year) |
| --- | --- | --- | --- | --- | --- | --- |
| pNFL (pg/mL)  [95% CI] | Good Outcome | 10.73  [7.72 to 13.74] | 8.24  [5.95 to 10.53] | 7.80  [5.21 to 10.39] | 7.80  [6.22 to 9.37] | 1.03  [-0.56 to 3.41] |
|  | Poor Outcome | 11.43  [5.31 to 17.55] | 13.97  [6.69 to 21.24] | 13.70  [6.75 to 20.66] | 15.03  [10.80 to 19.27] |  |
| pNFL (pg/mL) adjusted for T2LV  [95% CI] | Good Outcome | 16.58  [3.98 to 29.18] | 12.62  [8.80 to 16.44] | 11.06  [6.68 to 15.44] | 8.32  [6.44 to 10.21] | 0.91  [-0.42 to 3.29] |
|  | Poor Outcome | 14.57  [6.07 to 23.06] | 11.41  [4.24 to 18.58] | 10.41  [5.45 to 15.36] | 11.23  [6.86 to 15.60] |  |

Marginal means and 95% confidence intervals for plasma NFL at each timepoint in the good and poor outcome groups, following exclusion of the 29 serum samples. Estimates from a single model based upon distributions generated from 10,000 bootstrap replications. The overall difference in the rate of change of each dependent variable from baseline to 5 years (plus bias-corrected and accelerated 95% confidence intervals) from a separate model with time as a continuous variable is also reported in the final column. pNFL, plasma Neurofilament light; T2LV, T2 lesion volume.
